# Medicaid spending and utilization of gene and RNA therapies for rare inherited conditions

**DOI:** 10.1093/haschl/qxae051

**Published:** 2024-04-26

**Authors:** Ilina C Odouard, Jeromie Ballreich, Mariana P Socal

**Affiliations:** Department of Health Policy and Management, Johns Hopkins Bloomberg School of Public Health, Baltimore, MD 21205, United States; Department of Health Policy and Management, Johns Hopkins Bloomberg School of Public Health, Baltimore, MD 21205, United States; Department of Health Policy and Management, Johns Hopkins Bloomberg School of Public Health, Baltimore, MD 21205, United States

**Keywords:** gene therapy, RNA therapy, rare disease, Medicaid spending, Medicaid utilization

## Abstract

Gene and RNA therapies are promising treatments for many rare diseases. Pediatric populations that could benefit from these drugs are overrepresented among state Medicaid programs. Using Medicaid State Drug Utilization Data, we examined Medicaid spending and utilization of rare disease gene and RNA therapies. Between 2017 and 2022, the number of available gene and RNA therapies increased from 3 to 13, yearly Medicaid spending increased from $148.3 million to $879.7 million, and the number of yearly treatments (a proxy for number of patients) increased from 327 to 1638. Nearly all spending was attributed to spinal muscular atrophy (SMA) and Duchenne muscular dystrophy drugs. States participating in Medicaid pooled purchasing initiatives had 39% higher treatments per 100 000 enrollees with no differences in spending. Compared to states without a carve-out, states that carved SMA drugs out of managed Medicaid contracts had higher utilization (54%). Spending among carve-out states varied according to managed care enrollment, being higher for those with <80% of enrollees in managed care as compared with those with ≥80% of enrollees in managed care. This suggests that multi-state purchasing initiatives and managed care carve-outs can help increase access to gene and RNA therapies among Medicaid beneficiaries, but it is unclear if these strategies are effective at managing spending.

## Introduction

Gene and RNA therapies can transform the treatment of rare inherited conditions, but they usually have high costs. One of the first of such therapies, nusinersen (Spinraza; Biogen), was approved in 2016 to treat spinal muscular atrophy (SMA) and costs nearly $800 000 per year. Medicaid programs are key payers for rare inherited disease gene and RNA therapies because these drugs are often used in the pediatric population, which constitutes close to 50% of Medicaid and Children's Health Insurance Program (CHIP) beneficiaries.^1^ Approximately 20% of gene therapy spending in the next 10 years in the United States is expected to come from Medicaid.^2^

As overall Medicaid drug spending has increased, states have experimented with cost-containment strategies, including Medicaid pooling initiatives, preferred drug lists, and carve-outs to control spending. Three Medicaid pooling initiatives with a total of 29 participating states help states save an additional 3%–5% through supplemental rebates by placing drugs with additional rebates on preferred drug lists.^3^ Given that managed care organizations (MCOs) are contracted at a capitated rate by Medicaid programs to provide coverage for beneficiaries, some states have also begun carving certain high-cost drugs out of managed Medicaid contracts due to the complexity of setting reasonable capitation rates when those drugs are included.^4^ Some of these strategies have been applied to gene and RNA therapies already, as Medicaid directors are concerned about spending on these drugs.^5^ Starting in 2021, 11 states carved SMA drugs out from managed Medicaid, shifting the cost of those drugs to the fee-for-service program.^6,7^ The Centers for Medicare and Medicaid Innovation (CMMI) and some states are also exploring innovative payment models for gene therapies.^8^ This study examined Medicaid utilization and spending between 2017 and 2022 on gene and RNA therapies treating rare inherited conditions.

## Data and methods

### Data

The Food and Drug Administration (FDA) defines gene therapies as drugs that add or remove genetic material or modify gene expression.^9^ RNA therapies, which include messenger RNA, RNA interference, oligonucleotides, and antisense therapy, are also indicated to treat rare inherited conditions as they modify gene expression.^10^ We used several FDA databases—the National Drug Code (NDC) Directory,^11^ the approved cell and gene therapies database,^12^ the new drug approvals database,^13^ the orphan drug database,^14^ and the drug label database^15^—as well as FDA summary review documents and approval letters^16^ to identify all gene and RNA therapies treating rare inherited diseases approved between 2016 and 2022, and to collect NDCs, mechanisms of action, dosing regimen, indications, and target population for each drug. We obtained drug list prices (wholesale acquisition cost) from NAVLIN, a pharmaceutical pricing database.^17^

We identified the drugs by NDC in unsuppressed Medicaid State Drug Utilization Data from 2017 (the first year with data available for the drugs in our sample) to 2022 (the most recent available year). Medicaid State Drug Utilization Data records drug utilization of covered outpatient drugs in Medicaid programs in all 50 states and the District of Columbia eligible for manufacturer rebates under the Medicaid Drug Rebate Program; this excludes drugs purchased through the 340B program.^18^

Medicaid and CHIP enrollment data were obtained from Data.Medicaid.gov,^19^ and the percentage of enrollees in managed Medicaid was obtained from the Medicaid Managed Care Enrollment Report.^20^ States participating in Medicaid pooled purchasing initiatives (the National Medicaid Pooling Initiative, the Top Dollar Program, and the Sovereign States Drug Consortium) as of 2022 were identified from the National Conference of State Legislatures.^3^ States that carved SMA drugs out of managed Medicaid were identified from annual Medicaid budget survey reports by the Kaiser Family Foundation.^6,7^

### Outcome variables

We calculated the total pre-rebate (“gross”) Medicaid spending across all drugs and by indication, nationally and by state. We calculated gross spending per 1000 enrollees per state using annual spending on the sample drugs divided by the total number of Medicaid and CHIP enrollees in that state for each year. All spending figures were expressed in inflation-adjusted 2022 US dollars.^21^ We calculated the number of treatments administered per year (a proxy for number of patients treated) by dividing the number of prescriptions recorded in the Medicaid database in each year by the number of administrations required per the dosing information on the drug label for 1 year of treatment (for drugs with continuous dosing) and for 1 single treatment (for drugs with 1-time dosing). For each year and indication, we also calculated the number of states with utilization of at least 1 gene or RNA therapy.

### Statistical analysis

We performed multivariate regressions to test whether Medicaid spending (standardized per 1000 enrollees) and number of treatments (standardized per 100 000 enrollees) for gene and RNA therapies were associated with 4 key state characteristics: Medicaid expansion, participation in Medicaid pooled purchasing initiatives, having ≥80% of Medicaid enrollees in comprehensive managed care (80% was the median among the 50 states and the District of Columbia as of 2021, the most recent year for which managed-care enrollment data were available), and carving SMA drugs out of managed Medicaid contracts (this occurred in 2021 or 2022). We used a generalized linear model with the gamma family and log link due to the right-skewed nature of the data, which violates the normality assumption. As a sensitivity analysis, we varied the number of prescriptions assumed to constitute 1 year of treatment.

The study did not constitute human participants research because Medicaid data are provided at the drug level. Therefore, Institutional Review Board review was not sought.

### Limitations

This study had several limitations. First, spending figures represent gross spending before rebates, thus not representing actual costs to Medicaid. Medicaid rebates are calculated as the greater of 23.1% (or 17.1% for drugs with only pediatric indications) of the drugs’ list price or the highest rebate offered to other US payers.^22^ Inflationary or supplemental rebates individually negotiated between states and manufacturers independently or as part of pooled purchasing or value-based agreements may also apply, and are not captured in the data but could contribute to additional differences in spending between states.^22^ By not accounting for rebates, this study overestimated the actual Medicaid spending on gene and RNA therapies. Second, the data do not include utilization of drugs purchased through the 340B program. This likely does not substantially impact the findings, because manufacturers are not required to apply 340B discounts to orphan drugs; therefore, there is likely minimal 340B utilization of the drugs in this sample.^23^ Third, the data also did not account for beneficiary out-of-pocket costs and differences in coverage policies by state, which could influence spending and utilization; these were outside the scope of the study but represent important avenues for future investigations. Fourth, although most of the spending in this sample came from SMA and Duchenne muscular dystrophy (DMD) RNA therapies, these trends may not necessarily continue in the future as more gene therapies enter the market in other indications.

## Results

We identified 16 gene and RNA therapies to treat rare inherited conditions approved between 2016 and 2022 (Table S1); 13 of these drugs (81%) were recorded in the Medicaid data and constituted the final study sample. The 3 gene therapies not recorded in the data had been approved in the latter half of 2022.

Eight drugs (62%) were approved between 2016 and 2019 and 5 (39%) were approved between 2020 and 2022 (Table S2). Eleven drugs (85%) were RNA therapies with continuous dosing, while 2 (15%) were gene therapies with 1-time dosing. All drugs had orphan approvals and treated only 1 indication. Three conditions had more than 1 gene or RNA therapy available: DMD (*n* = 4 drugs), SMA (*n* = 3 drugs), and polyneuropathy of hereditary transthyretin-mediated amyloidosis (*n* = 3 drugs). The most common route of administration was intravenous infusion (*n* = 6 drugs) and other injections (*n* = 6 drugs), with 1 drug administered orally. Drugs were indicated for pediatric populations (*n* = 7 drugs), adult populations (*n* = 4 drugs), or both (*n* = 2 drugs). The mean (SD) annual treatment cost of drugs with continuous dosing was $585 495 ($171 714), while the 1-time dosing drugs cost $850 000 and $2 254 412, respectively.

**Figure 1.**
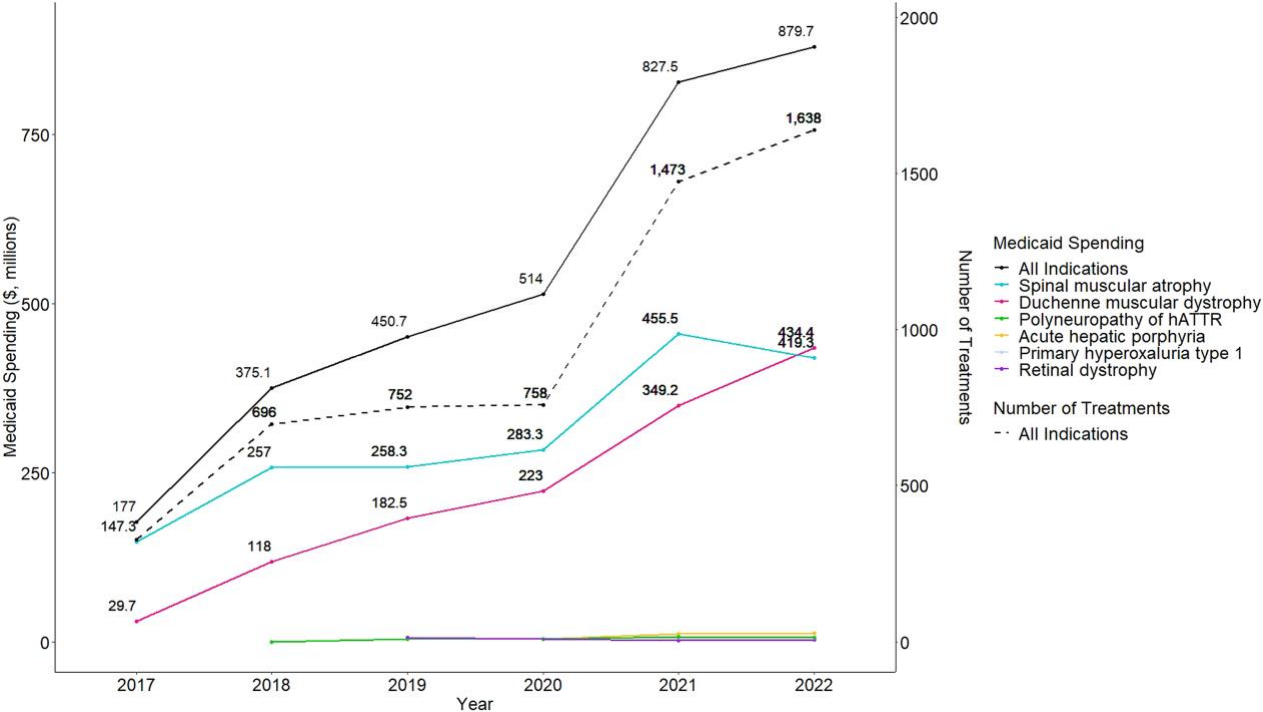
National gross Medicaid spending and utilization on gene and RNA therapies to treat rare inherited conditions, 2017–2022. Source: unsuppressed Medicaid State Drug Utilization Data (complete data without suppression for cells with <11 counts), 2017–2022. Spending does not include third-party spending such as patient out-of-pocket costs and other amounts reimbursed by non-Medicaid entities to pharmacies for which the state is not eligible for federal matching funds. Spending also does not account for rebates that may be provided by drug manufacturers to the Medicaid program. Number of treatments represents the number of full years’ worth of treatment administered, which approximates the number of patients treated. This was calculated by dividing the number of prescriptions recorded in the Medicaid database in each year by the number of administrations required per the dosing information on the drug label for 1 year of treatment (for drugs with continuous dosing) and for 1 single treatment (for drugs with 1-time dosing).

Between 2017 and 2022, the number of rare inherited disease gene and RNA therapies utilized in Medicaid increased from 3 to 13 and gross Medicaid spending on this group of drugs increased 5-fold from $177.0 million to $879.7 million nationally (growing from an average of $4.1 million to $17.2 million per state), and the number of treatments administered per year increased 4-fold from 327 to 1638 treatments nationally (growing from an average of 9.1 to 32.1 treatments per state). Spending was driven by drugs indicated for SMA (*n* = 3 drugs) and DMD (*n* = 4 drugs), which increased from $147.3 million to $419.3 million and $29.7 million to $434.4 million, respectively (Figure 1).

**Figure 2.**
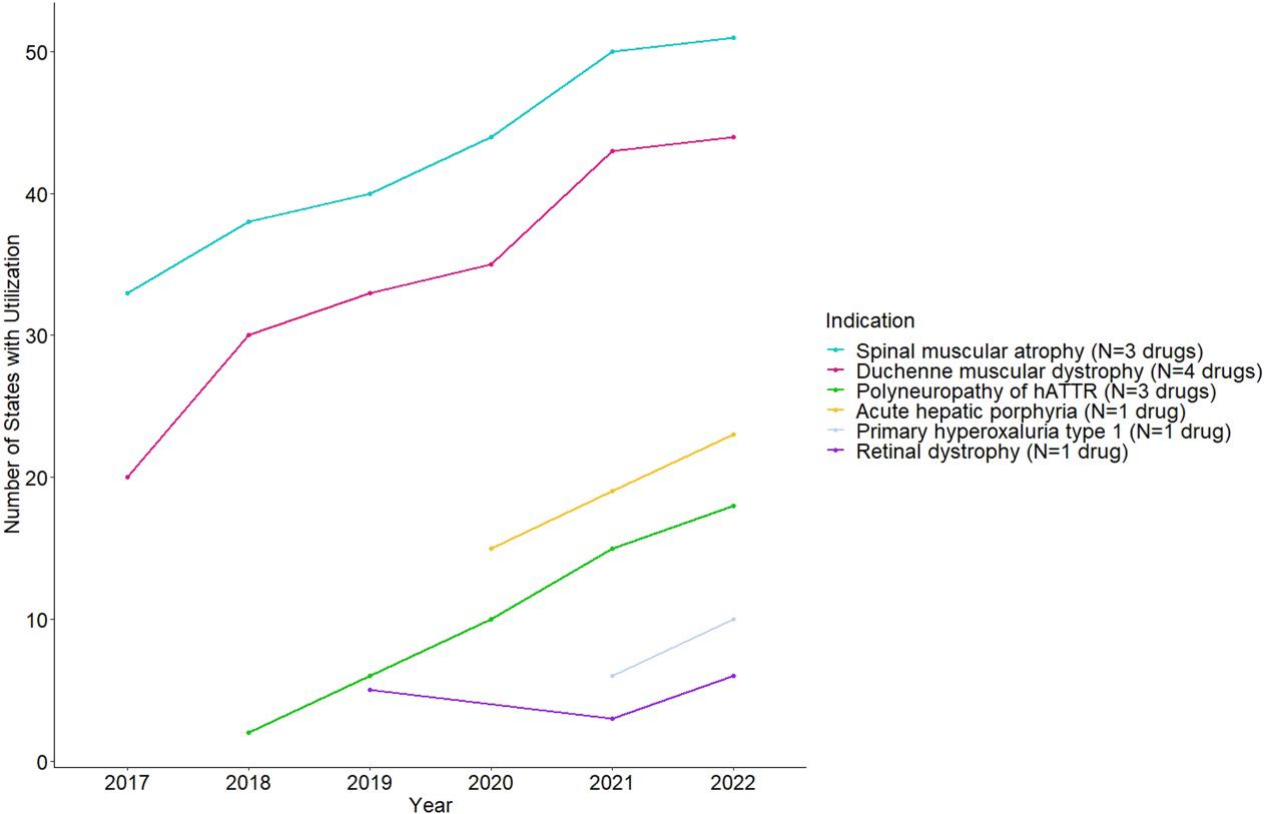
Diffusion of gene and RNA therapies by indication across states, 2017–2022. Source: unsuppressed Medicaid State Drug Utilization Data (complete data without suppression for cells with <11 counts), 2017–2022. For each indication, the number of states refers to the number of states that used at least 1 drug in that indication. The District of Columbia is included as a state. Abbreviation: hATTR, hereditary transthyretin-mediated amyloidosis.

Spinal muscular atrophy and DMD drugs were used in the highest number of states throughout the period compared with drugs in other indications. Thirty-three states used SMA drugs and 20 states used DMD drugs in 2017, the year the first drug in each of these indications was recorded in the Medicaid data. In contrast, the drugs in other indications were only used in 15 or fewer states in the first year of utilization. Despite differences between indications in the number of early adopter states, the pace of diffusion of these drugs across states consistently increased by an average of 2 to 4 states per year in most of the indications. In 2022, the last year of the study period, 51 states used at least 1 SMA drug and 44 states used at least 1 DMD drug, while between 6 (retinal dystrophy) and 23 (acute hepatic porphyria) states used drugs in the other indications (Figure 2).

**Figure 3.**
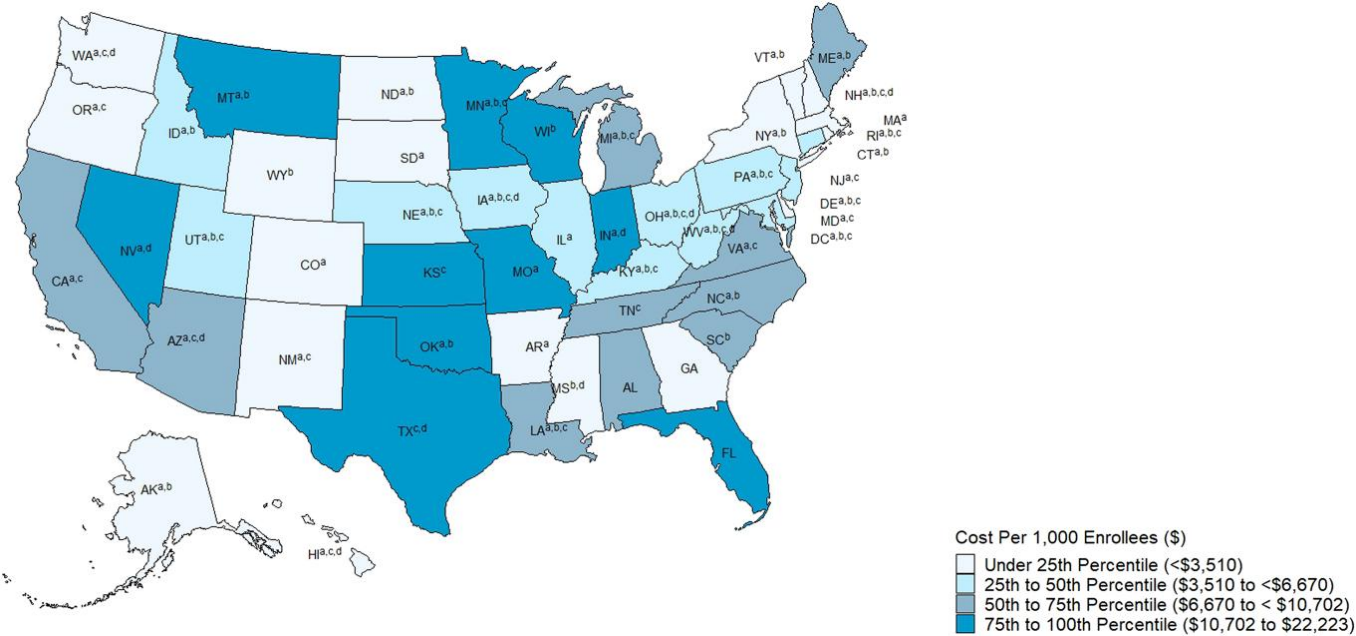
Cumulative gross spending per 1000 Medicaid and CHIP enrollees on gene and RNA therapies treating rare inherited conditions by state, 2017–2022. Source: unsuppressed Medicaid State Drug Utilization Data (complete data without suppression for cells with <11 counts), 2017–2022. Spending does not include third-party spending such as patient out-of-pocket costs and other amounts reimbursed by non-Medicaid entities to pharmacies for which the state is not eligible for federal matching funds. Spending also does not account for rebates that may be provided by drug manufacturers to the Medicaid program. ^a^Expanded Medicaid before or during the study period. ^b^Participated in a Medicaid pooled purchasing initiative as of 2022. ^c^Eighty percent or more of enrollees were in managed Medicaid as of July 2021. ^d^Carved SMA drugs out of MCO contracts during the study period. The Colorado Department of Health Care Policy & Financing announced that, on January 1, 2022, Colorado Medicaid implemented a value-based contract with Novartis for onasemnogene abeparvovec (Zolgensma; Novartis), a gene therapy for SMA. The agreement will allow the state to recoup part of the cost of the medication if desired health outcomes are not achieved over a 5-year time horizon after treatment.^24^ As of February 2023, Arizona also implemented a value-based payment agreement for onasemnogene abeparvovec, according to Secretary Becerra's report in response to Biden's executive order on lowering prescription drug costs.^25^ Abbreviations: CHIP, Children's Health Insurance Program; MCO, managed care organization; SMA, spinal muscular atrophy.

Median (IQR) cumulative spending per 1000 Medicaid and CHIP enrollees was $6670 (IQR, $3510–$10 702) (Figure 3). Seventy percent of states (7/10) that did not expand Medicaid, including Texas, Florida, Kansas, and Wisconsin, were between the 50th and 100th percentile (between $6670 and $22 223) of spending on these drugs per 1000 enrollees compared to 32% of states (13/41) that had expanded Medicaid. Sixty-five percent of states (17/26) with ≥80% of enrollees in managed Medicaid, 64% (7/11) of states with SMA drugs carved out of managed Medicaid, and 63% of states (19/30) that participated in Medicaid pooled purchasing initiatives had spending below the 50th percentile ($6670).

States that expanded Medicaid, a process which makes more low-income adults eligible for Medicaid coverage, had 23% (95% CI, 13%–48%; *P* < .01) lower spending on these drugs per 1000 enrollees compared to states that did not expand Medicaid after adjusting for other covariates (Table 1). The number of treatments per 100 000 enrollees was 34% lower in expanded states compared to non-expanded states (95% CI, 18%–51%; *P* < .001). States participating in pooled purchasing initiatives had 39% more treatments per 100 000 enrollees compared to states that did not participate in pooled purchasing (95% CI, 13%–72%; *P* < .001), but there was no difference in spending per 1000 enrollees between these states. States with ≥80% of enrollees in managed Medicaid did not significantly differ on spending and utilization compared with states with <80% of enrollees in managed Medicaid in the adjusted models, although the unadjusted model showed 21% lower spending among states with ≥80% of enrollees in managed Medicaid (95% CI, 4%–35%; *P* < .05). Carving SMA drugs out of managed Medicaid was associated with 54% more treatments per 100 000 enrollees (95% CI, 2%–40%; *P* < .001), all other things being equal. This did not significantly differ based on proportion of enrollees in managed care (exp. coef. 0.73, 95% CI 0.43–1.20; *P* < .05). Carveouts were associated with increased spending per 1000 enrollees by 71% among states with <80% of enrollees in managed care, (95% CI, 12%–168%; *P* < .05) but had no effect among states with ≥80% of enrollees in managed care (exp. coef. 1.02, 95% CI 0.76–1.37; *P* > .05). This means that, among states with a carve-out, spending was 50% lower in states with ≥80% of enrollees in managed care compared to states with <80% of enrollees in managed care (exp. coef. 0.50, 95% CI 0.32–0.79; *P* < .01). Varying the assumptions on number of prescriptions per year of treatment did not substantively change the results (Tables S3 and S4).

**Table 1.** Gene and RNA therapy differences in gross Medicaid spending per 1000 enrollees and treatments per 100 000 enrollees by state characteristic.

Variable	Medicaid spending per 1000 enrollees, exponentiated coefficient (95% CI)		Treatments per 100 000 enrollees, exponentiated coefficient (95% CI)	
	Unadjusted model	Adjusted model	Unadjusted model	Adjusted model
Medicaid expansion	0.71 (0.54, 0.90)**	0.67 (0.52, 0.87)**	0.71 (0.55, 0.91)**	0.64 (0.49, 0.82)***
Participation in Medicaid pooled purchasing initiative	0.93 (0.76, 1.14)	1.06 (0.86, 1.32)	1.18 (0.98, 1.44)	1.39 (1.13, 1.72)***
≥80% of enrollees in managed Medicaid	0.79 (0.65, 0.96)*	0.84 (0.67, 1.06)	0.91 (0.75, 1.10)	0.97 (0.78, 1.22)
SMA drugs carved out of MCOs	1.09 (0.86, 1.39)	1.71 (1.12, 2.68)*	1.08 (0.86, 1.37)	1.54 (1.02, 2.40)*
≥80% MCO × carve-out	—	0.60 (0.35, 0.99)*	—	0.73 (0.43, 1.20)
*n*	269^a^	269^a^	274	274

Abbreviations: MCO, managed care organization; SMA, spinal muscular atrophy.

Source: unsuppressed Medicaid State Drug Utilization Data (complete data without suppression for cells with <11 counts), 2017–2022. Spending does not include third-party spending such as patient out-of-pocket costs and other amounts reimbursed by non-Medicaid entities to pharmacies for which the state is not eligible for federal matching funds. Spending also does not account for rebates that may be provided by drug manufacturers to the Medicaid program. Number of treatments represents the number of full years’ worth of treatment administered, which approximates the number of patients treated. This was calculated by dividing number of prescriptions recorded in the Medicaid database in each year by the number of administrations required per the dosing information on the drug label for 1 year of treatment (for drugs with continuous dosing) and for 1 single treatment (for drugs with 1-time dosing). Unadjusted model results correspond to the outcomes (annual Medicaid spending per 1000 enrollees per state and annual treatments per 100 000 enrollees per state) regressed on each covariate individually. Adjusted model results correspond to the outcomes regressed on all covariates simultaneously. Results are presented as exponentiated coefficients (95% CIs) representing the multiplier of expected difference in the outcome. Regression models also controlled for year fixed effects with significant results, but those coefficients are not shown in the table. Statistical significance was set at alpha = .05. *P < .05, **P < .01, ***P < .001.

^a^Delaware was excluded from the Medicaid spending regressions for all years except for 2021 because it had $0 spending for those years, and gamma-family generalized linear models do not accept nonpositive values.

## Discussion

Gross Medicaid spending on rare inherited disease gene and RNA therapies increased 5-fold from 2017 to 2022, reaching $879.7 million (average of $17.2 million per state), with the number of rare disease gene and RNA therapies available and utilized in Medicaid increasing from 3 to 13 during the same period. Although the therapies in our sample treated 6 different conditions, SMA and DMD drugs comprised most of the spending and were used in the most states. There were also wide differences between states in spending per 1000 and treatments per 100 000 enrollees. Participating in pooled purchasing and carving out SMA drugs from managed Medicaid were both associated with higher utilization. Spending among carve-out states varied according to managed care enrollment, being higher for those with <80% of enrollees in managed care as compared with those with ≥80% of enrollees in managed care. Medicaid expansion was associated with both lower spending and utilization at the per-enrollee level.

Spinal muscular atrophy and DMD drugs drove most of the spending on this sample of drugs and were used in the highest number of states. This is likely in part because the SMA and DMD drugs are primarily used in the pediatric population, which constitutes 50% of the Medicaid population.^1^ In contrast, the other drugs in the sample are indicated either for adults or both adult and pediatric patients. A second reason for higher spending on SMA and DMD drugs is that there were multiple drugs in each of these indications during the period of this study, and they often treated complementary subpopulations. Together, the 3 SMA drugs cover all types of SMA,^26^ maximizing the size of the eligible patient pool. The 4 DMD drugs target 3 distinct types of DMD, representing at least 29% of patients.^27^ A third reason is that the list prices of SMA and DMD drugs were higher: the SMA and DMD RNA therapies cost around $800 000 annually, whereas the RNA therapies for other indications cost $500 000 or less. Given the overlap in subpopulations treated by the 3 SMA drugs, there could be unexpected increased costs if some patients receive more than 1 therapy, although several Medicaid programs guard against this risk by covering only 1 gene or RNA therapy per patient.^28^

Increasing spending on SMA and DMD gene and RNA therapies suggests that Medicaid programs are particularly vulnerable to new spending increases when confronted with high-cost therapies targeting pediatric populations. The percentage of all gene therapy spending in the United States allocated to rare diseases is expected to increase from 4.3% to 46.2% in the next 10 years, and annual Medicaid spending on gene therapies is expected to reach $5.4 billion, suggesting that new pediatric rare disease therapies could pose greater challenges to Medicaid budgets in the future.^2^ While only approximately half of states adopted the new SMA and DMD therapies initially, diffusion across states increased steadily to all states using SMA drugs and 44 states using DMD drugs by 2022, indicating that all states have been confronted with the budgetary challenges posed by at least 1 of these drugs. Spinal muscular atrophy and DMD drugs may cease to be the dominant gene and RNA therapies in Medicaid in the future as the number of other available therapies continues to increase. Newly approved gene therapies for sickle cell disease, for example, are expected to increase Medicaid spending by $30 million per state in the first year.^29^ In addition, there are likely more eligible patients who have not yet been treated by existing therapies due to access barriers,^28,30^ suggesting that spending could also continue to grow among existing therapies.

Differences in spending per 1000 enrollees and treatments per 100 000 enrollees among this group of drugs could have been due to differences in state-level characteristics. Our analysis finds that, for the study sample of drugs, states with Medicaid expansion had 33% lower spending per 1000 enrollees and 34% fewer treatments per 100 000 enrollees. This is expected given that Medicaid expansion provides coverage to more low-income adults,^31^ but likely does not substantially increase the Medicaid-eligible pediatric population, which is the main target of the drugs examined in this study. This suggests that states that have expanded Medicaid are better able to weather financial challenges posed by high-cost pediatric drugs because they are better able to spread costs and utilization among a larger population of enrollees, of whom the newly eligible adults under the Affordable Care Act are largely covered by federal funds.^31^

Differences in payment approaches may have also explained some of the variation in spending and utilization between states. Pooled purchasing was associated with 39% higher utilization of gene and RNA therapies, suggesting that these initiatives have increased patient access through use of either single- or multi-state preferred drug lists linked with supplemental rebates.^3^ Medicaid pooled purchasing initiatives provide savings to states through supplemental rebates that are not reflected in Medicaid gross spending data. Therefore, it is expected that no difference in gross spending should be present between states participating in those initiatives compared with states that did not. Higher utilization suggests that pooled purchasing may be an effective method of increasing access to gene and RNA therapies. This could also suggest that CMMI's new cell and gene therapy access model, which will negotiate the terms of an outcomes-based agreement for Medicaid purchasing of sickle cell gene therapies,^32^ could also have an access-enabling effect like the Medicaid pooled purchasing initiatives.

States that carved SMA drugs out of managed Medicaid had 54% more treatments per 100 000 enrollees, suggesting that shifting the cost of SMA therapies to the fee-for-service program increased access. However, the effect of the carve-out on spending differed based on the proportion of enrollees in managed care: states with <80% of enrollees in managed Medicaid had higher spending compared to states with ≥80% enrollees in managed Medicaid. Eight out of 11 states in the sample that carved SMA drugs out of managed care had ≥80% of enrollees in managed Medicaid, suggesting that the carve-out was more appealing to states with high reliance on managed care. This analysis suggests that states leveraging managed care to cover a high proportion of enrollees may benefit from carving out the SMA drugs rather than incorporating those costs into managed-care capitation rates more than states with a lower proportion of enrollees covered by managed care.

The differences in gross spending levels between states could have also been, in part, attributed to pay-over-time arrangements in some states. It is possible that some states had value-based agreements allowing them to pay over time, given that 22 states have approved waivers from the Centers for Medicare and Medicaid Services that allow them to enter into value-based agreements.^33^ At least 2 states (Colorado and Arizona) are known to have implemented a value-based based agreement for onasemnogene abeparvovec as of 2022 and 2023.^24,25^ Although these particular agreements may have occurred too late to observe an effect in this analysis, it is possible that other states had similar agreements for drugs in this sample earlier in the period that were not publicly announced, which might have influenced these states’ annual gross spending levels. Finally, differences in both spending and utilization levels could also be, in part, driven by differences in the mix of drugs used between states.

## Conclusion

Substantial increases in national spending and utilization of gene and RNA therapies since 2017 support the rationale for new payment models to both mitigate budgetary challenges to Medicaid programs while providing access to innovative new therapies. Our findings suggest that pooled purchasing initiatives that use preferred drug lists and supplemental rebates are associated with higher utilization of these drugs, and not higher gross spending. Carving SMA drugs out of managed Medicaid contracts was associated with higher utilization, but also higher gross spending among states with fewer than 80% of enrollees in managed Medicaid. States that have expanded Medicaid may be better equipped to spread new and increasing spending on gene and RNA therapies, particularly for rare pediatric diseases, among a larger population of enrollees. Further research should examine the effectiveness of Medicaid strategies, such as pooled purchasing, preferred drug lists, and carve-outs, in reducing net spending and expanding access to these therapies for all patients who can benefit.

## Supplementary Material

qxae051_Supplementary_Data
