# Supplementary material for: Medicaid spending and utilization of gene and RNA therapies for rare inherited conditions
Source: Health Aff Sch. 2024 Apr 26;2(5):qxae051. doi: 10.1093/haschl/qxae051 (PMC11104525; doi:10.1093/haschl/qxae051)
Supplement: qxae051_Supplementary_Data [file qxae051_supplementary_data.zip › Appendix_Gene and RNA Medicaid_HASch_R1.docx]

**MEDICAID SPENDING AND UTILIZATION OF GENE and rna THERAPIES for RARE INHERITED CONDITIONS**

**Supplementary Material**

**eTable 1. Detailed drug characteristics.**

| **Gene Therapy Name (Brand)** | **Manufacturer** | **Approval Date** | **Mechanism of action** | **Dosing** | **Annual or One-Time Treatment Cost^a^** | **Indication** | **Therapeutic Alternatives** | **Population** |
| --- | --- | --- | --- | --- | --- | --- | --- | --- |
| Givosiran (Givlaari)^b^ | Alnylam | 11/20/2019 | Small interfering RNA (siRNA)^c^ | Monthly subcutaneous injection | $496,500 | Acute hepatic porphyria (AHP) | Hemin | Adults |
| Eteplirsen (Exondys 51)^b^ | Sarepta | 9/19/2016 | Antisense oligonucleotide^c^ | Weekly IV infusions | $748,800 | Duchenne muscular dystrophy (DMD) | Glucocorticoid treatments (prednisone, deflazacort) | Primarily pediatric |
| Golodirsen (Vyondys 53) | Sarepta | 12/12/2019 | Antisense oligonucleotide^c^ | Weekly IV infusions | $748,800 |  |  |  |
| Viltolarsen (Viltepso) | NS Pharma | 8/12/2020 | Antisense oligonucleotide^c^ | Weekly IV infusions | $733,000 |  |  |  |
| Casimersen (Amondys 45) | Sarepta | 2/25/2021 | Antisense oligonucleotide^c^ | Weekly IV infusions | $748,800 |  |  |  |
| Patisiran (Onpattro)^b^ | Alnylam | 8/10/2018 | Small interfering RNA (siRNA)^c^ | IV infusions every 3 weeks | $503,229 | Polyneuropathy of hereditary transthyretin-mediated amyloidosis(hATTR) | Transthyretin tetramers stabilizers (tafamidis and diflunisal) | Adults |
| Inotersen (Tesgedi) | Ionis Pharmaceuticals | 10/5/2018 | Antisense oligonucleotide^c^ | Weekly subcutaneous injection | $481,388 |  |  |  |
| Vutrisiran (Amvuttra) | Alnylam | 6/13/2022 | Small interfering RNA-GaINAc conjugate^c^ | Subcutaneous injection every 3 months | $477,404 |  |  |  |
| Lumasiran (Oxlumo)^b^ | Alnylam | 11/23/2020 | Small interfering RNA (siRNA)^c^ | Subcutaneous injection every 1-3 months | $333,423 | Primary hyperoxaluria type 1 (PH1) | Pyridoxine, potassium citrate | Pediatric and adult |
| Voretigene neparvovec-rzyl (Luxturna)^b^ | Spark Therapeutics | 12/19/2017 | Adeno-associated virus vector-based gene therapy^d^ | One-time subretinal injection per eye | $850,000 | RPE65-related retinal dystrophy | None | Pediatric and adult |
| Nusinersen (Spinraza)^b^ | Biogen | 12/23/2016 | Antisense oligonucleotide^c^ | Intrathecal injection every 4 months (after loading dose) | $799,425 | Spinal muscular atrophy (SMA) | None | Primarily pediatric |
| Onasemnogene abeparvovec-xioi (Zolgensma) | Novartis | 5/24/2019 | Adeno-associated virus vector-based gene therapy^d^ | One-time IV infusion | $2,254,412 |  |  | Pediatric (<2 years) |
| Risdiplam (Evrysdi) | Genentech | 8/7/2020 | Splicing modifier^c^ | Daily oral solution | $369,673 |  |  | Primarily pediatric (≥2 months) |

Sources/Notes: Product Package Inserts, FDA Approval Letters, NAVLIN Data (EVERSANA’s Global Pricing & Market Access Database).

^a^Represents the estimated annual treatment cost for drugs with continuous dosing or one-time treatment cost for drugs with one-time dosing from the NAVLIN database using the wholesale acquisition cost (WAC).

^b^First gene or RNA therapy in indication.

^c^RNA therapy.

^d^Gene therapy.

**eTable 2.** **Characteristics of gene and RNA therapies treating rare inherited conditions with utilization in the Medicaid program, 2017-2022**

| **Characteristic** | **Value** |
| --- | --- |
| Approval year,^a^ N(%) |  |
| 2016-2019 | 8 (61.5%) |
| 2020-2022 | 5 (38.5%) |
| Drug type,^a,b^ N(%) |  |
| RNA therapy | 11 (84.6%) |
| Gene therapy | 2 (15.4%) |
| Dosing regimen,^a^ N(%) |  |
| Continuous | 11 (84.6%) |
| One-time | 2 (15.4%) |
| Route of Administration,^a,c^ N(%) |  |
| IV infusion | 6 (46.2%) |
| Other injectable | 6 (46.2%) |
| Oral | 1 (7.7%) |
| Indication,^a^ N(%) |  |
| Drug has only 1 indication | 19 (100%) |
| Drug only treats orphan indications | 19 (100%) |
| Indication,^a^ N(%) |  |
| Duchenne muscular dystrophy (DMD) | 4 (30.8%) |
| Polyneuropathy of hereditary transthyretin-mediated amyloidosis (hATTR) | 3 (23.1%) |
| Spinal muscular atrophy (SMA) | 3 (23.1%) |
| Acute hepatic porphyria (AHP) | 1 (7.7%) |
| Primary hyperoxaluria type 1 (PH1) | 1 (7.7%) |
| RPE65-related retinal dystrophy | 1 (7.7%) |
| Population treated,^a,d^ N (%) |  |
| Primarily pediatric | 7 (53.8%) |
| Adult | 4 (30.8%) |
| Pediatric and adult | 2 (15.4%) |
| Therapeutic alternatives,^e^ N (%) |  |
| Alternatives are available and include gene therapies | 10 (76.9%) |
| Non-gene therapy alternatives are available | 2 (15.4%) |
| No therapeutic alternatives are available | 1 (7.7%) |
| Price per treatment,^f^ Mean (SD) |  |
| One-time dosing (N=2 drugs) | $1,552,206 ($993,069)  Range $850,000 - $2,254,412 |
| Continuous dosing drugs (N=11 drugs) | $585,495 ($171,714)  Range $333,423 - $799,425 |

^a^Source: FDA drug labels database

^b^RNA therapies were antisense oligonucleotides and small interfering RNAs. Gene therapies were viral vector-based gene therapies.

^c^Other injectables include subcutaneous (N=4 drugs), intrathecal (N=1), and subretinal (N=1) injections.

^d^Primarily pediatric refers to drugs explicitly indicated for pediatric populations or characterized in the label as primarily treating pediatric populations given the nature of the disease.

^e^Source: UpToDate

^f^Source: NAVLIN Data (EVERSANA’s Global Pricing & Market Access Database). Price per treatment reflects the wholesale acquisition cost for a single dose of treatment (for one-time dosing drugs) or for a 12-month treatment course (for continuous dosing drugs) and does not account for rebates or discounts that may be provided by the drug manufacturer to the Medicaid program.

**eTable 3.** Assumptions on number of prescriptions per treatment.

| **Drug** | **Indication** | **Dosing frequency, according to label** | **Assumed number of prescriptions per year of treatment** | | | **Rationale** |
| --- | --- | --- | --- | --- | --- | --- |
|  |  |  | ***Low*** | ***Base*** | ***High*** |  |
| Givosiran (Givlaari) | Acute hepatic porphyria (AHP) | Monthly subcutaneous injection | 12 | 12 | 12 | Assumed one Rx per dose |
| Eteplirsen (Exondys 51) | Duchenne muscular dystrophy (DMD) | Weekly infusions | 52 | 52 | 52 | Assumed one Rx per dose |
| Golodirsen (Vyondys 53) |  | Weekly infusions | 52 | 52 | 52 | Assumed one Rx per dose |
| Viltolarsen (Viltepso) |  | Weekly infusions | 52 | 52 | 52 | Assumed one Rx per dose |
| Casimersen (Amondys 45) |  | Weekly infusions | 52 | 52 | 52 | Assumed one Rx per dose |
| Patisiran (Onpattro) | Polyneuropathy of hereditary transthyretin-mediated amyloidosis(hATTR) | Infusion every 3 weeks | 17 | 17 | 17 | Assumed one Rx per dose |
| Inotersen (Tesgedi) |  | Weekly subcutaneous injection | 12 | 12 | 12 | Assumed one Rx per month given injections are self-administered |
| Vutrisiran (Amvuttra) |  | Subcutaneous injection every 3 months | 4 | 4 | 4 | Assumed one Rx per dose |
| Lumasiran (Oxlumo) | Primary hyperoxaluria type 1 (PH1) | Subcutaneous injection every 1-3 months | 6 | 6 | 6 | Assumed one Rx per dose, assuming on average doses every 2 months |
| Voretigene neparvovec-rzyl (Luxturna) | RPE65-related retinal dystrophy | One injection per eye | 1 | 2 | 2 | Assumed 2 Rx (1 per eye) as base and high case, and 1 Rx as low case in case some patients are only treated in one eye |
| Nusinersen (Spinraza) | Spinal muscular atrophy (SMA) | Loading: 4 doses within first 3 months  Maintenance: intrathecal injection every 4 months | 2 | 3 | 4 | Assumed 2-4 Rx needed per patient per year given higher number of doses in first year compared to maintenance, and possibility of patients starting treatment mid-year |
| Onasemnogene abeparvovec-xioi (Zolgensma) |  | One-time infusion | 1 | 1 | 1 | Assumed one Rx per dose |
| Risdiplam (Evrysdi) |  | Daily oral solution | 12 | 12 | 12 | Assumed one Rx per month given daily dosing |

Sources/Notes: Product Package Inserts. Rx: prescription.

**eTable 4.** Sensitivity analysis varying assumptions on number of prescriptions per treatment.

| **Variable** | **Treatments per 100,000 enrollees, exponentiated coefficient (95% CI)** | | |
| --- | --- | --- | --- |
|  | **Low case^a^** | **Base case** | **High case^a^** |
| Medicaid expansion | 0.64 (0.50, 0.82)*** | 0.64 (0.49, 0.82)*** | 0.63 (0.48, 0.81)*** |
| Participation in Medicaid pooled purchasing initiative | 1.37 (1.12, 1.68)** | 1.39 (1.13, 1.72)*** | 1.43 (1.15, 1.76)*** |
| ≥80% of enrollees in managed Medicaid | 0.96 (0.77, 1.20) | 0.97 (0.78, 1.22) | 0.99 (0.79, 1.25) |
| SMA drugs carved out of MCOs | 1.51 (1.01, 2.35)* | 1.54 (1.02, 2.40)* | 1.60 (1.05, 2.51)* |
| ≥80% MCO x Carve-out | 0.75 (0.45, 1.23) | 0.73 (0.43, 1.20) | 0.69 (0.40, 1.15) |
| N | 274 | 274 | 274 |

Source/notes: unsuppressed Medicaid State Drug Utilization Data (complete data without suppression for cells with fewer than 11 counts), 2017-2022. Adjusted model results correspond to the outcomes (annual Medicaid spending per 1,000 enrollees per state and annual treatments per 100,000 enrollees per state) regressed on all covariates simultaneously. Regression models also controlled for year fixed effects with significant results, but those coefficients are not shown in the table.

^a^Low case and high case refer to scenarios with a lower number of treatments and higher number of treatments. Number of treatments is calculated by dividing the number of prescriptions by the number of administrations of the drug required per year, according to the drug label. Therefore, a lower number of treatments will result from assuming a higher number of administrations (prescriptions) per treatment. The low and high scenarios vary the number of prescriptions per treatment for Spinraza and Luxturna; all other drugs have consistent assumptions across scenarios. Low case: assumes 4 prescriptions = 1 treatment for Spinraza and 2 prescriptions = 1 treatment for Luxturna. Base case: assumes 3 prescriptions = 1 treatment for Spinraza and 2 prescriptions = 1 treatment for Luxturna. High case: assumes 2 prescriptions = 1 treatment for Spinraza and 1 prescription = 1 treatment for Luxturna.
